# Effectiveness of transitioning from omalizumab to dupilumab in chronic spontaneous urticaria patients with inadequate response to omalizumab^☆^

**DOI:** 10.1016/j.waojou.2025.101098

**Published:** 2025-08-07

**Authors:** Koremasa Hayama, Mana Ito-Watanabe, Hideki Fujita

**Affiliations:** a Division of Cutaneous Science, Department of Dermatology, Nihon University School of Medicine, Tokyo, Japan; b Center for Allergy, Nihon University Itabashi Hospital, Tokyo, Japan

**Keywords:** Chronic spontaneous urticaria, Omalizumab, Dupilumab, Interleukin-4, Immunoglobulin E

## Abstract

Dupilumab is effective for chronic spontaneous urticaria (CSU), but its clinical benefit of switching to dupilumab in omalizumab-resistant CSU remains unclear.

This prospective pilot study evaluated the effectiveness of dupilumab in 12 CSU patients whose Urticaria Control Test (UCT) scores remained <12 after receiving at least 4 doses of omalizumab.

Patients were transitioned to dupilumab after inadequate response to omalizumab. UCT scores were assessed at baseline, and 1 and 4 months after starting dupilumab. Laboratory parameters including eosinophil and basophil counts, total IgE, C-reactive protein, and anti-thyroid peroxidase antibody levels were also evaluated.

The mean UCT score at the initiation of dupilumab was 6.5 ± 2.2, improving to 8.4 ± 3.0 at 1 month (P = 0.016) and 8.1 ± 3.9 at 4 months (P = 0.045), compared to 4.9 ± 3.1 before omalizumab treatment. Three patients achieved UCT ≥12 after switching to dupilumab. No significant differences were found in clinical characteristics or laboratory markers between effective and ineffective groups. Interestingly, 3 patients who returned to omalizumab after inadequate response to dupilumab achieved UCT ≥12.

Dupilumab may improve disease control in a subset of omalizumab-resistant CSU patients. Further studies are needed to identify which patients benefit most from switching therapies.

## Introduction

Chronic spontaneous urticaria (CSU) is an inflammatory condition characterized by recurrent wheals and angioedema lasting over 6 weeks, significantly impacting quality of life due to itching and daily disruptions.^1^ Biologic therapies are considered for antihistamine-resistant patients. Omalizumab, a monoclonal antibody against free immunoglobulin E (IgE), was the only approved biologic for CSU until February 2024 in Japan and remains the only widely internationally approved biologic, showing efficacy in about 80% of patients after 6 months.^2^^,^^3^ Th2 cytokines activate mast cells and eosinophils in CSU, indicating benefits from targeting these molecules.^4^, ^5^, ^6^ Dupilumab, which targets interleukin (IL)-4/13 receptor, is effective for CSU.^7^ Clinical trials, however, did not show significant improvements in the Urticaria Activity Score over 7 days (UAS7) and Urticaria Control Test (UCT) after 24 weeks of dupilumab treatment in CSU patients intolerant or incomplete responders to omalizumab.^7^ Although not statistically significant, the dupilumab arm showed a numerically higher disease control rate than placebo, which prompted us to investigate its real-world effectiveness.

## Methods

We conducted a pilot study at Nihon University Itabashi Hospital involving CSU patients with UCT scores of less than 12 after receiving 4 doses of 300 mg of omalizumab every 4 weeks, with the administration intervals extended to 6–8 weeks in some patients. The patients were switched to dupilumab, starting with 600 mg followed by 300 mg biweekly, and UCT scores were assessed at baseline (start of dupilumab) and 1 and 4 months post-switch. All patients concomitantly used second-generation antihistamines at twice the standard doses approved in Japan. There were no patients taking oral steroids or cyclosporine. White blood cell counts, serum IgE, and C-reactive protein (CRP) levels were measured before omalizumab, at baseline, and after 1 and 4 months. Statistical analysis assessed correlations between these factors and treatment responses. The Ethics Committee of Nihon University Itabashi Hospital approved this study (RK- 240813–8).

## Results

**Fig. 1:**
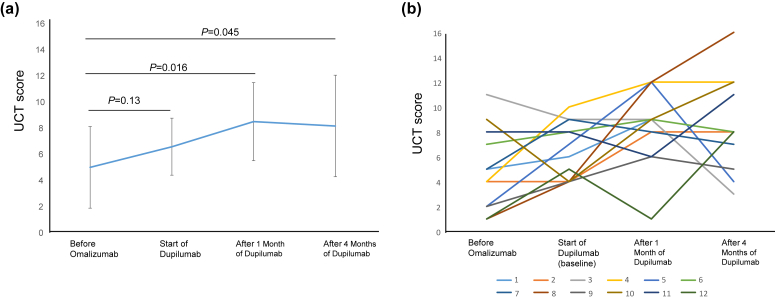
(a) Time course of Mean UCT score. Mean ± standard deviation, (b) Time courses of UCT scores of each patient.

Twelve CSU patients who were incomplete responders to omalizumab (Table 1) included 4 males and 8 females, with a mean age of 41.6 ± 19.5 years. They received an average of 21.6 ± 20.7 doses of omalizumab before switching to dupilumab. The mean IgE level was 635.3 ± 1049.7 IU/mL at the start of omalizumab, increasing to 1046.4 ± 1701.1 IU/mL at the switch (Supplementary Table 1). The mean UCT score before omalizumab was 4.9 ± 3.1, rising to 6.5 ± 2.2 at the dupilumab start, with this increase not significant. The score further rose to 8.4 ± 3.0, showing significant improvement compared to that before omalizumab (P = 0.016; Fig. 1a). Four months post-switch, the mean UCT score was 8.1 ± 3.9, which remained significant compared to that before omalizumab therapy (P = 0.045; Fig. 1a). On the other hand, 4 out of 12 patients showed decreased UCT scores compared to baseline before starting dupilumab.

**Table 1:** Data on patients switched from omalizumab to dupilumab.

Patient	Age (years)	Sex	AD	asthma	Number of Oma Doses	Duration of Oma (months)	UCT Score Before Oma	UCT Score at baseline	UCT Score After 1 Month of Dup	UCT Score After 4 Months of Dup
1	17	F	–	–	6	7	5	6	9	3
2	72	M	+	–	18	16	4	4	8	8
3	19	F	–	–	18	25	11	9	9	3
4	16	F	+	–	20	20	4	10	12	12
5	38	F	+	–	25	30	2	7	12	4
6	79	M	–	–	3	3	7	8	9	8
7	40	M	–	–	85	82	5	9	8	7
8	55	M	–	–	4	5	1	4	12	16
9	39	F	–	–	22	36	2	4	6	5
10	30	F	–	+	29	29	9	4	9	12
11	50	F	–	–	13	13	8	8	6	11
12	32	F	–	+	16	24	1	5	1	8
Mean (±SD)	40.6 ± 19.5				21.6 ± 20.7	24.2 ± 20.1	4.9 ± 3.0	6.5 ± 2.2	8.4 ± 3.0	8.1 ± 3.9

Patients who achieved a UCT score ≥12 after 4-month dupilumab treatment are highlighted in blue (effective group)

Patients were classified based on their UCT score after 4 months of dupilumab treatment. Three patients with a score ≥12 were classified as the effective group, while the other 9 were classified as ineffective (Table 1; Fig. 1b). Two of the 3 effective patients had a UCT score equal to 12, and 1 did not reach the minimum important difference of 3 points. No significant differences were found between the 2 groups for UCT scores (P = 0.67), IgE levels (P = 0.37), eosinophil counts (P = 0.60), basophil counts (P = 0.60), anti-thyroid peroxidase (TPO)-antibody levels (P = 0.13), CRP levels (P = 0.28), and serum IgE changes (P = 0.72) before dupilumab treatment (Supplementary Table 2). In addition, the history of asthma or atopic dermatitis was comparable between the 2 groups (P = 0.52).

Three dupilumab-failure patients resumed 300 mg of monthly omalizumab and achieved a UCT score ≥12 1 month later, which continued for over 3 months.

## Discussion

Our findings suggest that dupilumab may provide short-term symptom relief for some omalizumab-resistant CSU patients. However, their clinical improvements were limited, because improvement in mean UCT sore during this treatment did not reach minimum important difference of 3 points. While 3 of 12 patients achieved a UCT score ≥12, 4 patients showed exacerbation. These results indicate the variability of treatment responses and the need for endotype-based approaches. Dupilumab targets IL-4 and IL-13, which contribute to pathophysiology of CSU by upregulating IgE production and FcεRI expression and through IgE-independent mechanisms such as neuronal sensitization.^6^, ^7^, ^8^ Indeed, serum IgE levels decreased in all patients 4 months after starting dupilumab. This may explain the effectiveness observed in some omalizumab non-responders depending on the contribution of the pathways in each patient. In addition, one-third of dupilumab non-responders attained disease control after switching back to omalizumab, suggesting that IL-4/13 blockade may reduce IgE responsiveness by downregulating IgE production and FcεRI expression and/or Th2-driven inflammation. However, consistent with previous studies, there was no significant difference in IgE reduction rates between the effective and ineffective groups. This suggests a need for further research on the association between IgE dynamics and clinical effectiveness.

No significant differences were found between effective and ineffective groups in baseline UCT scores, serum IgE levels, eosinophil and basophil counts, CRP levels, or asthma and atopic dermatitis history. While anti-TPO antibody is proposed as a marker for type IIb autoimmune urticaria rather than type I autoallergic urticaria,^1^ it did not affect treatment outcomes either in this study. Although specific endotypes of CSU suitable for dupilumab treatment are still to be elucidated, CSU patients with atopic dermatitis and/or asthma can benefit from this agent because dupilumab is also approved for these conditions. Larger cohort studies are necessary to investigate underlying pathophysiological endotypes eligible for dupilumab treatment.

Limitations of this study include a small sample size, single-center design, lack of UAS7 data, limited biomarker evaluation, and relatively short-term administration of dupilumab. Further, many patients discontinued dupilumab due to insufficient effectiveness and costs.

Switching from omalizumab to dupilumab showed limited effectiveness in omalizumab-resistant UCT patients. Further research is needed to identify which patients can benefit most from this drug switch.

## Abbreviations

CRP, C-reactive protein; CSU, chronic spontaneous urticaria; IgE, immunoglobulin E; IL, interleukin; UAS7, Urticaria Activity Score over 7 days; TPO, thyroid peroxidase; UCT, Urticaria Activity Score.

## Availability of data and material

The data that support the findings of this study are available on request from the corresponding author, Fujita H. The data are not publicly available because they contain information that could compromise the privacy of research participants.

## Authors' contributions

All authors contributed equally to the conception, design, data collection, analysis, and drafting of this manuscript. All authors have reviewed and approved the final version of the manuscript.

## Ethics

This study was approved by the Ethics Committee of Nihon University Itabashi Hospital (RK- 240813–8).

## Funding

This work was supported by JSPS KAKENHI Grant Number 22K08393.

## Declaration of competing interest

Hayama K has received study grants or honoraria for speaking engagements from Boehringer-Ingelheim, Kaken Pharmaceutical, Kyorin, Kyowa-Kirin, Maruho, Mitubishi-Tanabe, Novartis, Sanofi, and Taiho.

Ito-Watanabe M has no conflicts of interest.

Fujita H has received study grants or honoraria for speaking engagements from Boehringer-Ingelheim, Kaken Pharmaceutical, Kyorin, Kyowa-Kirin, Maruho, Mitubishi-Tanabe, Novartis, Sanofi, and Taiho.

## Footnotes

Appendix A Supplementary data to this article can be found online at https://doi.org/10.1016/j.waojou.2025.101098.
