# Supplementary material for: Effectiveness of transitioning from omalizumab to dupilumab in chronic spontaneous urticaria patients with inadequate response to omalizumab
Source: World Allergy Organ J. 2025 Aug 7;18(8):101098. doi: 10.1016/j.waojou.2025.101098 (PMC12355504; doi:10.1016/j.waojou.2025.101098)
Supplement: Multimedia component 1 [file mmc1.pdf]

Patient	IgE Level before OMA (IU/ml)	IgE Level at baseline (IU/ml)	Change in IgE between pre-OMA and baseline (%)	WBC at baseline (/μL)	Eo count at beseline (/μL)	Baso count at baseline (/μL)	CRP at baseline (mg/L)	Anti-TPO-IgG (IU／mL)	IgE Level after 4 Months of Dup (IU/ml)	Change in IgE after 4 Months of Dup (%)	WBC After 4 Months of Dup (/μL)	Eo Count After 4 Months of Dup (/μL)	Baso Count After 4 Months of Dup (/μL)	CRP After 4 Months of Dup (mg/L)
1	496	966	48.7	9100	136.5	27.3	2.21	≦9	335	-65.3	8700	121.8	26.1	3.05
2	3900	6584	40.8	4440	79.92	8.88	6.56	20	3991	-39.4	5100	30.6	20.4	9.90
3	166	300	44.7	3200	41.6	9.6	0.02	10	167	-44.3	3600	61.2	21.6	0.14
4	631	949	33.5	4300	129	51.6	0.13	≦9	596	-37.2	5560	389.2	38.92	0.14
5	449	1027	56.3	7300	445.3	58.4	0.11	≦9	285	-72.2	6700	227.8	60.3	0.30
6	n.d	79	n.d	5300	233.2	31.8	1.07	18	53	-32.9	6600	224.4	52.8	0.56
7	562	655	14.2	8000	48	32	0.58	15	283	-56.8	10100	121.2	30.3	2.11
8	149	146	-2.1	6600	112.2	13.2	2.19	≦9	46	-68.5	6600	112.2	13.2	0.22
9	202	415	51.3	6900	0	0	0.35	12	155	-62.7	5000	10	0.0	0.47
10	33	163	79.8	6200	80.6	37.2	0.08	≦9	72	-55.8	7200	129.6	0.0	0.08
11	109	449	75.7	9400	47	56.4	0.13	218	149	-66.8	7500	142.5	0.0	2.06
12	291	824	64.7	8500	93.5	17.0	17.89	≦9	139	-83.1	8900	89	0.0	15.65
Mean (±SD)	635.3±1049.7	1046.4±1701.1	46.1±23.4	6603.3±1966.2	120.6±113.3	28.6±18.8	0.27±0.49	26.7±58.0※	522.6±1056.1	-57.1±15.0	6796.7±1769.1	138.3±98.4	22.0±20.0	0.29±0.47

Baso : Basophil, CRP: C reactive protein, Dup: Dupilumab, Eo: Eosinophil, IU: international unit, Oma: Omalizumab, SD: standard deviation, TPO: thyroid peroxidase, WBC: white blood cell

Normal range for Baso Count 6.6～77.4 /μL, Eo Count 70～440 /μL, IgE: ≤170 IU/mL, WBC 3,300～8,600/μL

※Values below the detection limit were imputed as half the detection limit (4.5).

S. Table 1: Laboratory Data on patients switched from omalizumab to dupilumab.
