# Supplementary material for: Effectiveness of transitioning from omalizumab to dupilumab in chronic spontaneous urticaria patients with inadequate response to omalizumab
Source: World Allergy Organ J. 2025 Aug 7;18(8):101098. doi: 10.1016/j.waojou.2025.101098 (PMC12355504; doi:10.1016/j.waojou.2025.101098)
Supplement: Multimedia component 2 [file mmc2.pdf]

	Effective group (n=3)	Ineffective group (n=9)	*P value*
**UCT before Dup administration ,mean±SD**	**6.0±2.8**	**6.7±1.9**	**0.69＊**
**Serum IgE before Dup administration (IU/ml), mean±SD**	**419.3±374.6**	**1255.4±1907.0**	**0.37＊**
**Peripheral blood eosinophil count before Dup administration (/μL), mean±SD**	**107.3±20.1**	**125.0±130.0**	**0.60＊**
**Peripheral blood basophil count before Dup administration (/μL), mean±SD**	**34.0±15.8**	**26.8±19.3**	**0.60＊**
**Serum CRP before Dup administration (mg/L), mean±SD**	**0.80±0.98**	**3.36±5.47**	**0.28＊**
**Change in IgE after 4 Months of Dup (%), mean±SD**	**-53.8±12.9**	**-58.2±15.5**	**0.73＊**
**Anti-TPO antibody before Oma (IU/ml) , mean±SD**	**4.5±0.0♯**	**34.1±65.3♯**	**0.13＊**
**History of Atopic dermatitis or asthma**	**2**	**3**	**0.52†**

CRP: C reactive protein, Dup: Dupilumab, SD: standard deviation, TPO: thyroid peroxidase, UCT: urticaria control test
＊: Mann–Whitney U test
†: Fisher's exact test
♯:Values below the detection limit were imputed as half the detection limit (4.5).

S. Table 2: Comparison of effective and ineffective groups
